# Supplementary material for: Novel Insights into the Differences in Nutrition Value, Gene Regulation and Network Organization between Muscles from Pasture-Fed and Barn-Fed Goats
Source: Foods. 2022 Jan 28;11(3):381. doi: 10.3390/foods11030381 (PMC8834483; doi:10.3390/foods11030381)
Supplement: Supplementary file 1 [file foods-11-00381-s001.zip › foods-1576384-Supplementary.pdf]

Table S1. Primers used in this study.

Target gene	primer sequence (5′−3′)	Target gene	primer sequence (5′−3′)
β-actin-F	AGCCTTCCTTCCTGGGCATGGA	FASN-F	TTTCAGACCTTGGCCTCGAC
β-actin-R	GGACAGCACCGTGTTGGCGTAA	FASN-R	TGTGTGGAGTTCGTCAGCTC
LPIN-F	AACCGTTACTACAGCTGGGC	SCD-F	CTTCTCCGGTACGCTGTTGT
LPIN-R	GCAGCTGCTTCGTTATGCTC	SCD-R	ATGGTCTTGTCGTAAGGGCG
PLIN2-F	CACCCTCCTGTCCAACATCC	HMGCS1-F	CCACAGGAAATGCCAGACCT
PLIN2-R	TAAAGGAGGCAGCATTGCGA	HMGCS1-R	CCCACGAAGTCCTCGTTCAA
LDLR-F	ATGTGACTCTTTGCGAGGGG	FDFT1-F	GGACTTTGGTGCTGAGTCCC
LDLR-R	GTCCCTGATGGAGTTGCACA	FDFT1-R	GTGCTGGAGTGGGGCTG
PCYTIA-F	ACCCTGACACCAGAGTTCCT	MSTRG.57988.4-F	TGCGGTAGCGTAGTTTCCTT
PCYTIA-R	GAGCAAACATGCCAGCTTCC	MSTRG.57988.4-R	TAGCGCCTACTCGTCAGGAT
ACSS2-F	GGGCGAATGCCTCTACTGTT	MSTRG.39270.1-F	CCCCGACTGGAAAAATCCGA
ACSS2-R	GCCTCATGATTTTCCCTGAGC	MSTRG.39270.1-R	CCAACAGACGTCACCAAACG
ACSL5-F	AAACCAGCCCTACAGATGGC	MSTRG.41627.1-F	ACCCAATCAGTCCACTACAAGC
ACSL5-R	GCCAACCAAGCTGTCTTGTG	MSTRG.41627.1-R	AGTTTTAGGCAGTCAGGGTGG
MEF2C-F	ACCACCACCCCTTCGAGATA	MSTRG.17648.1-F	CCTGCTCCGGCTTCAACTAT
MEF2C-R	GGAGTGGAATTCGTTCCGGT	MSTRG.17648.1-R	TGGAGCCCCCGATATAGCAT
MSTN-F	CGCCTGGAAACAGCTCCTAA	MSTRG.13856.1-F	TTGTCATCCACCCAACCTGG
MSTN-R	TCGCTGCTGTCATCTCTCTG	MSTRG.13856.1-R	GGCCTTGGATTAGGCTTGGT
MYH7B-F	ACTGAGAGTGCTGACAAGGC	MSTRG.14111.2-F	AGGCCGAACAGGCAGATTTC
MYH7B-R	GTACTCATTCCCCACACGCA	MSTRG.14111.2-R	TTACAAGCCCTTTGGCCTCC
CHPT1-F	ACTGTCTTTATTGGCCCAGGT	MSTRG.19078.1-F	CGTGTGATAACAGCCCCCTT
CHPT1-R	ATTTGCAGGCACAAAGCACT	MSTRG.19078.1-R	TTGTCTTGGCACCCCCAAAT
PPARA-F	CGGCTGAAGCTGGTGTATGA	MSTRG.57988.1-F	AGCAGCAGGACACAGGATTC
PPARA-R	CAACGGAAAGGCACTTGTGG	MSTRG.57988.1-R	GGAGCTTGGGTAATTGGGCT
DHCR7-F	ACATCTTCCGGGTGACCAAC		
DHCR7-R	CTTGCTGTGGTGTTTCTGCC		

Table S3. Physiological and chemical characters of the muscles.

Items	Feeding model	
	Pasture-fed（n=3）	Barn-fed（n=3）
pH	6.898 ± 0.078^{A}	5.930 ± 0.071 ^{B}
Shear force(N/cm)	37.720 ± 1.887	37.780 ± 2.881
Flesh color	29.400 ± 0.207 ^{A}	27.980 ± 0.405 ^{B}
Electrical conductivity (S/m)	1.063 ± 0.042 ^{A}	1.450 ± 0.085 ^{B}
Cooked meat rate (%)	59.200 ± 2.307 ^{a}	47.770 ± 1.859 ^{b}
Water content （ g/100g）	77.233±0.767	74.900±1.290
Crude ash （ g/100g）	1.040±0.060	0.930±0.025
Crude protein （ g/100g）	21.500±0.100	19.840±0.783
Crude fat （ g/100g）	1.100±0.058 ^{a}	4.767±1.141 ^{b}

Note: a, b indicates $p < 0.050$, A, B indicates $p < 0.010$.

Table S4. Amino acid content of meat (longissimus lumborum) of black goats.

Amino acids	Feeding condition	
	Pasture-fed（n=3）	Barn-fed（n=3）
Asp (mg/100 g)	1.920±0.026	1.840±0.046
Thr (mg/100 g)	0.940±0.006	0.910±0.029
Ser (mg/100 g)	0.793±0.013	0.783±0.018
Glu (mg/100 g)	3.010±0.104	2.960±0.097
Gly (mg/100 g)	0.883±0.019 [a]	1.177±0.090 [b]
Ala (mg/100 g)	1.077±0.003 [A]	1.163±0.015 [B]
Cys (mg/100 g)	0.157±0.015	0.170±0.020
Val (mg/100 g)	1.007±0.007	0.973±0.027
Met (mg/100 g)	0.360±0.044	0.303±0.067
Ile (mg/100 g)	0.993±0.003 [A]	0.900±0.015 [B]
Leu (mg/100 g)	1.733±0.007 [a]	1.630±0.036 [b]
Tyr (mg/100 g)	0.637±0.024	0.650±0.035
Phe (mg/100 g)	0.840±0.006	0.810±0.025
Lys (mg/100 g)	1.897±0.012	1.803±0.055
His (mg/100 g)	0.733±0.009	0.720±0.050
Arg (mg/100 g)	1.313±0.007	1.330±0.006
Delicious amino acids (mg/100 g)	4.930±0.131	4.800±0.142
Essential amino acid (mg/100 g)	8.503±0.012	8.050±0.258
Total amino acids (%)	18.293±0.131	18.087±0.352

Note: a, b indicates $p < 0.050$, A, B indicates $p < 0.010$. Delicious amino acids = Asp+ Glu; Essential amino acid = Thr+ Val+ Met+ Ile+ Leu

Table S5. Mineral content of meat (longissimus lumborum) of black goats.

Element	Feeding condition	
	Pasture-fed（n=3）	Barn-fed（n=3）
P (mg/kg)	1980.667±66.333	1660.667±117.516
K (mg/kg)	3800.333±40.251	2876.667±366.865
Na (mg/kg)	532.667±32.895	846.333±184.194
Ca (mg/kg)	50.217±1.084	72.533±13.125
Mg (mg/kg)	260.333±8.172 [a]	204.000±10.408 [b]
Cu (mg/kg)	0.649±0.063	0.594±0.069
Fe (mg/kg)	17.910±1.761	17.753±0.850
Mn (mg/kg)	0.162±0.012 [a]	0.217±0.001 [b]
Zn (mg/kg)	34.620±1.343 [A]	25.880±0.841 [B]
Se (mg/kg)	0.097±0.005	0.127±0.012
Pb (mg/kg)	0.003±0.001	0.006±0.001
Cd (mg/kg)	0.001±0.000	0.000±0.000
Cr (mg/kg)	0.009±0.002	0.029±0.009
As (mg/kg)	0.002±0.000 [a]	0.004±0.000 [b]
Hg (mg/kg)	0.001±0.000	0.001±0.000
Ni (mg/kg)	0.006±0.002	0.011±0.002

Note: a, b indicates $p < 0.050$, A, B indicates $p < 0.010$.

Table S6. Serum biological indexes of the goats.

Items	Feeding condition	
	Pasture-fed（n=36）	Barn-fed（n=45）
ALT (U/L)	30.250 ± 0.665 ^{a}	26.760 ± 1.138 ^{b}
AST (U/L)	104.700 ± 6.884	94.780 ± 3.485
TP (g/L)	67.070 ± 1.050	66.700 ± 0.856
ALB (g/L)	29.170 ± 0.294 ^{a}	28.220 ± 0.313 ^{b}
GLB (g/L)	37.900 ± 1.131	38.470 ± 0.867
DBIL (μmol/L)	3.333 ± 0.296	3.882 ± 0.277
TBIL (μmol/L)	5.992 ± 0.501	6.960 ± 0.479
IBIL (μmol/L)	2.658 ± 0.224	3.078 ± 0.210
ALP (U)	474.500 ± 101.200	337.000 ± 45.410
Urea (mmol/L)	4.461 ± 0.139	4.803 ± 0.175
Crea (μmol/L)	31.080 ± 0.975	31.730 ± 0.949
UA (μmol/L)	8.583 ± 0.846	10.040 ± 0.777
TG (mmol/L)	0.471 ± 0.036 ^{A}	0.348 ± 0.017 ^{B}
CHOL (mmol/L)	2.381 ± 0.067 ^{A}	3.087 ± 0.113 ^{B}
HDL_C (mmol/L)	0.902 ± 0.024	0.982 ± 0.036
LDL_C (mmol/L)	0.783 ± 0.033 ^{A}	1.134 ± 0.049 ^{B}
GLU (mmol/L)	3.546 ± 0.163	3.856 ± 0.119
Ca (mmol/L)	6.720 ± 0.052 ^{A}	6.063 ± 0.114 ^{B}
Fe (mmol/L)	28.480 ± 1.148	28.350 ± 1.238
Mg (mmol/L)	0.841 ± 0.011	0.817 ± 0.022
P (mmol/L)	2.205 ± 0.107	2.114 ± 0.086
A/G	0.793 ± 0.026	0.752 ± 0.019
AST/ALT	3.497 ± 0.217	3.824 ± 0.225
D/T	0.558 ± 0.010	0.555 ± 0.007

Note: a, b indicates $p < 0.05$, A, B indicates $p < 0.01$.
